# The Carboxyl Functionalized UiO-66-(COOH)_2_ for Selective Adsorption of Sr^2+^

**DOI:** 10.3390/molecules27041208

**Published:** 2022-02-11

**Authors:** Yuan Gao, Yinhai Pan, Zihan Zhou, Quanzhi Tian, Rongli Jiang

**Affiliations:** 1 School of Chemical Engineering and Technology, China University of Mining & Technology, Xuzhou 221116, China; y.gao@cumt.edu.cn (Y.G.); pan043032@cumt.edu.cn (Y.P.); TB19040017B2@cumt.edu.cn (Z.Z.); 2 National Engineering Research Center of Coal Preparation and Purification, China University of Mining and Technology, Xuzhou 221116, China; tianqz0502@foxmail.com

**Keywords:** metal-organic framework, adsorption mechanism, competing ions

## Abstract

Efficient and selective removal of ^90^Sr is an important process for the safe use of nuclear energy. Herein, we investigate and assess the Sr^2+^ adsorption properties of a metal-organic framework UiO-66-(COOH)_2_ functionalized by non-bonded carboxylic groups. This MOF is an exciting class of free carboxylic functionalized MOFs that combine chemical stability with gas sorption, dye elimination, and conductivity. Specifically, we show that uniformly distributed carboxyl and water stability make it accessible for loading Sr^2+^ without structural changes. The FTIR spectroscopy, PXRD analysis, XPS, and SEM-EDS studies show excellent stability as well as the strong affinity between -COOH active site and Sr^2+^. This strong coordination interaction guarantees a high adsorption capacity of 114 mg g^−1^ within 5 h (pH 5 and 298 K). Combined kinetic and thermodynamic studies show that the surface complexation is strong chemisorption and cost-effective spontaneous process (ΔG = −5.49 kJ mol^−1^~−2.16 kJ mol^−1^). The fact that UiO-66-(COOH)_2_ not only possesses a high adsorption capacity, but also enables selectivity to Sr^2+^ in the presence of similar radius ions Na^+^ and K^+^, prefigures its great potential for the practical treatment of radioactive Sr^2+^ in polluted water.

## 1. Introduction

Nuclear energy, due to its low-carbon and high-efficiency nature, is widely used in electric power, military, and industrial applications nowadays. However, the use of nuclear energy brings radioactivation pollution inevitably [1]. ^90^Sr, as representative radioactive contamination with strong beta-gamma/beta emitters, is of particular concern for its long half-life (*t_1/2_*~28.8 years). This element is one major contributor to radioactivity and radiation and is highly toxic and hazardous to human health and the environment. In practice, the amount of Sr in nuclear waste streams is estimated to be ~4.2%, with much of the rest being non-radioactive components (e.g., sodium, potassium), which has chemical similarity, are always accompanied [2]. Therefore, one thorny issue existing is that the radioactivation species need to be selectively captured in the presence of competing ions. It is thus not supervised that designing an effective and selective extraction method of radiation in the presence of competing species is a significant challenge. Physio-chemical processes such as precipitation, liquid–liquid extraction, ion exchange, and adsorption have been used for the treatment of waste solutions [3,4,5,6]. Among types of methods, using solid-state adsorbents such as silicotitanates, metal sulfide, geopolymers, and zeolites have shown degrees of successful cost-effectiveness and selective removal of Sr in aqueous [7,8,9,10].

One emerging solid-state material is metal-organic frameworks (MOFs), featuring adjustable structure, simple synthesis, and high porosity, which are regarded as promising candidates in the field of selective adsorption and separation [11,12,13,14,15]. The importance of two factors justifies MOFs towards the adsorption of particular metal ions in the solution. The first is the chemical or physical interactions between positivity charge metal ions in solution and free electron pairs on ligand functional groups, and the other one returns to a requirement that the radius of metal ions matches up with the pore size. Grafting functional groups (e.g., -SO_3_H, -CO_2_H) and incorporating anions (e.g., [NH_2_(CH_3_)_3_]^+^) in pores can efficiently remediate cationic ions [16,17,18]. Although some work highlights the opportunity of MOFs for radioactive remediation, more detailed investigations are required into more water-stable adsorbents, the adsorption behaviors, and simultaneously competitive adsorption [2,19,20,21,22]. Generally, the kinetic and thermodynamic behaviors can give insights into the adsorption capacity and selectivity, as the equilibrium and thermal effect play a key role in the binding affinity. Thus, to design a selective adsorbent, we must first understand the mechanisms between the adsorbents and adsorbates.

UiO-66 features cub octahedral nodes Zr_6_O_4_(OH)_4_, which enable 12-fold connections to adjacent organic linkers, giving rise to high stability and the appropriate pore structure [23,24,25]. It is believed that carboxyl functional groups can not only reduce the surface charge of the adsorption material, but also provide abundant sites for cationic nuclides [26]. Therefore, we chose 1,2,4,5-benzene tetracarboxylic acid (H_4_btec) to construct UiO-66-(COOH)_2_, which inherit a classical UiO 3D framework with tetrahedral and octahedral cages [27]. It is noteworthy that two carboxylate arms of H_4_btec play the role of linkers, while the two remaining are non-bonded. These free carboxylic acid groups are uniformly distributed throughout the MOF making them readily accessible for binding with cations without structure collapse [28]. UiO-66-(COOH)_2_, its features of gas sorption, dyes elimination, conductivity, and good water stability, have been respectively assessed [27,29,30,31,32]. Here, we further expanded the application of UiO-66-(COOH)_2_ in radioactive contamination removal. We discovered that this carboxyl functionalized MOF can adsorb Sr^2+^, and explained the adsorption mechanism based on the results of the kinetic, thermodynamic, and characterization studies. We also evidenced that it showed high adsorption capacity and selectivity even in the presence of disturbing ions, which makes it a good candidate in real-life applications.

## 2. Materials and Instruments

### 2.1. Synthesis of UiO-66-(COOH)_2_

Unless stated otherwise, all chemicals were commercially available (Sigma-Aldrich, St. Louis, MI, USA; Shanghai Trading Co Ltd., Shanghai, China, >99% pure) and used as received. The water-stable UiO-66-(COOH)_2_ was synthesized according to the literature [33]. Briefly, in a round-bottom flask equipped with a reflux condenser and magnetic stirrer, 1,2,4,5-benzene tetracarboxylic acid (H_4_btec) (2.54 g, 10 mmol) and zirconium tetrachloride (ZrCl_4_) (2.43 g, 10.4 mmol) was dispersed in distilled water (60 mL) at room temperature under stirring and then heated under reflux (~100 °C) for 24 h to yield a powder product. The sample was dried under a dynamic vacuum at 70 °C to yield the final product.

### 2.2. Characterizations

Infrared spectra (4000–400 cm^−1^, resol. 0.5 cm^−1^) were recorded on a Varian 660 Fourier-transform infrared (FTIR) spectrometer using KBr pellets and the transmission technique. Powder X-ray diffraction (PXRD) measurements were carried out on a Bruker AXS D8 Advance Diffractometer using Cu-Kα radiation (λ = 0.154 nm, 35 kV, 40 mA). The data were collected from 5° to 60° with a turning speed of 2.0°/min. Thermogravimetric analysis (TGA) and derivative thermogravimetry (DTG) was performed using a NETZSCH Jupiter^®^ STA 449F3 instrument. The measurements were done under air (20 mL·min^−1^) at 35–800 °C with a speed of 10 K·min^−1^. The elemental analysis and surface states of the samples were analyzed by X-ray photoelectron spectroscopy (XPS), which were carried out on a Thermo Fisher spectrometer (ESCALAB 250Xi, Thermo Fisher, Auburn, AL, USA) using a monochromatized Al Ka radiation (*hv* = 1486.6 eV). Brunauer-Emmett-Teller (BET) specific surface area was investigated by the N_2_ adsorption-desorption method at 77 K on an ASAP 2020. The samples were activated at 300 °C overnight under a vacuum. The scanning electron microscope (SEM) and energy-dispersive X-ray (EDX) analyses were performed on a Zeiss Sigma 300 scanning electron microscope. The concentration of residual Cs^+^ and Sr^2+^ in aqueous was analyzed by an inductively coupled plasma optical emission spectrometer (ICP-OES). The zeta potentials of geopolymer samples were measured on a Zetasizer NANO-ZS (Malvern Panalytical Ltd., Worcestershire, UK) in a solution state.

### 2.3. Adsorption Test

Typically, 20 mg adsorbents and 20 mL solutions containing Sr^2+^ with appropriate concentration were added in a vial at the appropriate temperature and pH value. The pH was adjusted with 0.1 M negligible volume of HNO_3_ and NaOH. The equilibrium adsorption capacity of U(VI) (q_e_), removal efficiency (%), and distribution coefficient *K_d_* (mL g^−1^), was calculated as follows:

(1) $$\%\;\mathrm{Removal}=\frac{\left(C_{o}-C_{e}\right)\times 100}{C_{o}}$$

(2) $$\mathrm{qe}=\frac{\left(C_{o}-C_{e}\right)\times V}{m}$$

(3) $$K_{d}=\frac{\left(C_{o}-C_{e}\right)\times V}{C_{o}\times m}$$

where *C_o_* and *C_e_* are the initial and equilibrium concentrations of Sr^2+^ (mg/L), respectively, V is the liquid phase volume (L), and m is the amount of adsorbent (g).

## 3. Results and Discussion

**Figure 1:**
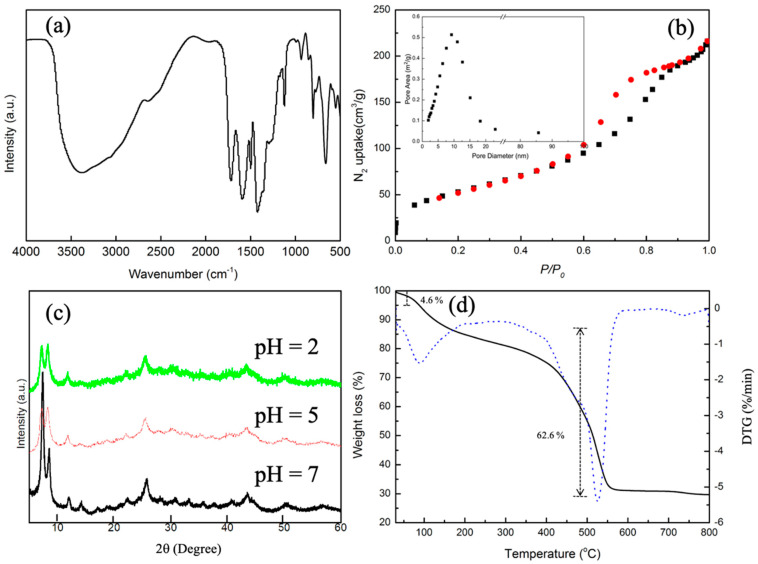
The FTIR spectrum (**a**), the surface area and pore size (insert) (**b**), PXRD patterns under various pH 2, 5, 7 (**c**), and TGA (lines) and DTG (dots) curves (**d**) of pristine UiO-66-(COOH)_2_.

The FTIR spectra of UiO-66-(COOH)_2_ featuring around 1700 cm^−1^ indicated that this material is abundant in carboxylic groups, providing the active sites in capturing metal ions (Figure 1a). The broad absorption band in the range 3100–3500 cm^−1^ is assigned to ν(O-H) of -COOH as well as water molecules. Together with N_2_ adsorption analysis (Figure 1b) showing an appropriate surface area of 194 cm g^−1^ and pore size of 6.8 nm, this MOF met the basic selective adsorption condition. The larger pore size is generated from the defection under acidic conditions [34]. Even with the existence of this defection, the chemical stability of the pristine MOF was confirmed by PXRD (see Figure 1c), where the characteristic peaks and degree of crystallinity remained when the immersed sample in solutions of various pH values (2, 5, and 7). Meanwhile, Figure 1d shows the TGA and DTG curves for the synthesized MOFs, and provide the information of solvent content and thermal stability of the framework. The first weight loss of 4.6% at 85 °C associated with one broad DTG peak, which corresponds to the removal of solvent molecules, indicates that no structural changes occur within the framework. The second weight loss of 62.6% from 85 to 550 °C and the appearance of a DTG peak above 500 °C is caused by the collapse of the structure. This thermal stability provides the possibility to study the adsorption performance under high temperatures. Combined with the fact that UiO-66-(COOH)_2_ possess 3D architecture with mesoporous cages, and the carboxyl groups are uniformly distributed in the framework, we hypothesized that chemical interaction will occur between Sr^2+^ and the -COOH active sites.

To study the possibility of selective adsorption, we must firstly optimize adsorption conditions and explore the mechanism in the absence of competing ions. The pH ranges do not affect the domination species of Sr^2+^, but the surface potential of MOFs changed with solution pH. Adjusting the pH value range of 2–7, the adsorption capacity and the pH values after adsorption were listed in Table S1. Except for the initial condition of pH 5, the solution pH was significantly reduced after adsorption. This indicated that proton exchanges occurred between the carboxyl groups and metal ions during the adsorption process [35,36]. By comprehensive consideration of pH effect on the adsorption capacity, the pH was fixed at 5 for further adsorption experiments.

**Figure 2:**
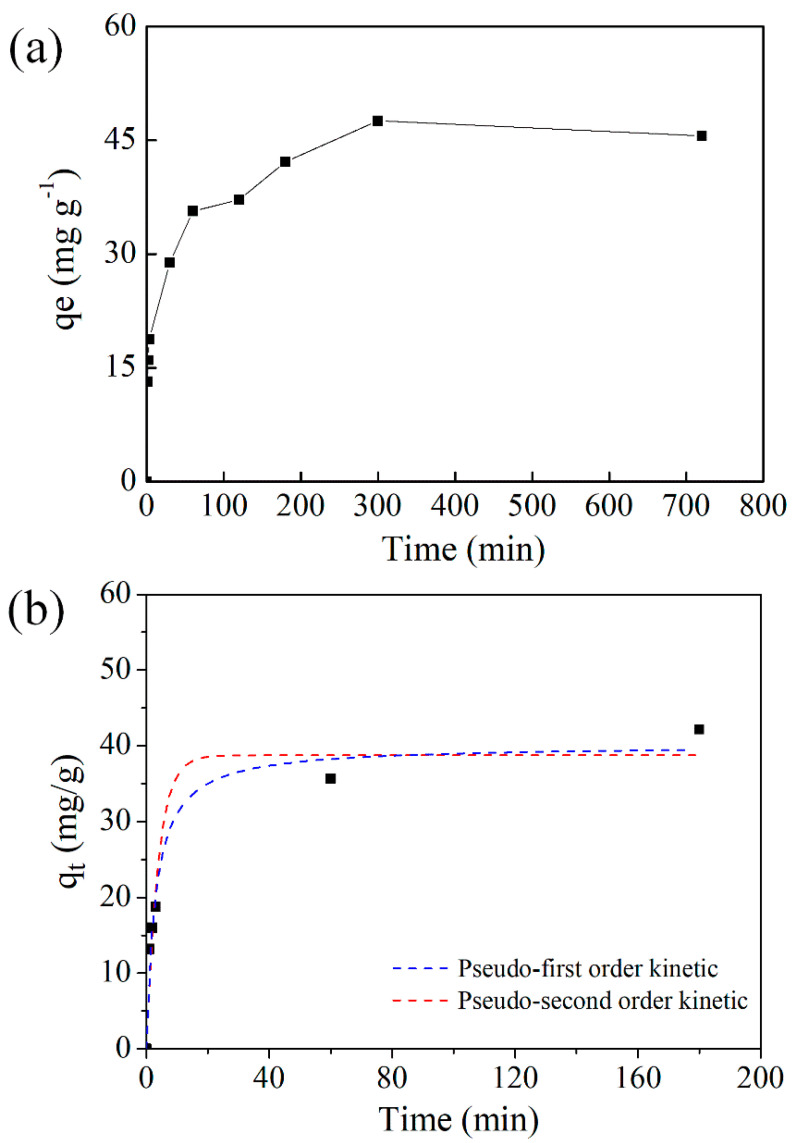
(**a**) Effect of contact time on Sr^2+^ onto UiO-66-(COOH)_2_ (Adsorption test: initial concentration 100 mg L, pH = 5.0, T = 293 K); (**b**) Kinetic studies of Sr^2+^ onto UiO-66-(COOH)_2_.

A preliminary study was done on the sorption amount of Sr^2+^ at increasing contact time, which is an important indicator of the feasibility of an adsorbent and the result was depicted in Figure 2a. The sorption amount reached a plateau in 2 h for Sr^2+^ with the value of approximately 40 mg g^−1^. It is known that the presence of carboxyl groups increases the defect concentration of the UiO-66 structure, producing a negative charge in acidic conditions, [28,37,38] therefore electrostatic attractions exist and contribute to a fast equilibrium adsorption process. While the zeta-potential value before and after adsorption were −31.7 and −33 mV, respectively (see Table S2), indicating the absence of the electronic interaction with cationic at room temperature. Therefore, the adsorption process involved a slow diffusion and relatively long equilibrium time. When prolonging the adsorption time, the capacity remained unchanged, indicating the high stability of adsorbents and the strong interactions with Sr^2+^. To achieve maximum removal efficiency and understand the adsorption mechanism, all further experiments were done at a contact time of 5 h under pH 5.0.

Subsequently, pseudo-first and pseudo-second-order models have been first applied to simulate the adsorption data (see Figure 2b and Table 1) to figure out the adsorption mode. The pseudo-first-order and pseudo-second-order models are present as below.

Pseudo-first-order:

(4) $$q_{t}=q_{e}\left(1-e^{-k_{1}t}\right)$$

Pseudo second-order:

(5) $$q_{t}=\frac{q_{e}^{2}k_{2}t}{1+q_{e}k_{2}t}$$

where *q_t_* and *q_e_* (mmol g^−1^) are the amounts of Sr^2+^ adsorbed onto samples at time *t* and equilibrium, respectively; *k_1_, k_2_* are the rate constants (min^−1^) of the pseudo-first-order and pseudo-second-order equations, respectively.

The pseudo-second-order model can describe the adsorption process more accurately than the pseudo-first-order model, because of its higher values of R-squared (R^2^) statistics and more similarity of calculated *q_e_* to experimental *q_e._* This suggested that chemical sorption might control the overall rate constant of each sorption process.

To obtain information about Sr^2+^ affinity as well as the distribution of the active sites on the adsorbent surface, we conducted the adsorption isotherm experiments by adjusting the initial concentrations of Sr^2+^ in the range of 20–250 ppm from 313 to 353 K at pH 5 and the contact time of 5 h. The models are described as follows [39]

Langmuir model:

(6) $$q_{e}=q_{m}\frac{bC_{e}}{1+bC_{e}}$$

Freundlich model:

(7) $$q_{e}=K_{f}C_{e}^{\frac{1}{n}}$$

where *q_e_* and *q_m_* (mg g^−1^) are the amounts of metal ions adsorbed onto the samples and the monolayer adsorption capacity, respectively. *b* is the constant related to the free energy of adsorption and *K_f_* is a constant indicative of the relative sorption capacity (mg g^−1^).

**Figure 3:**
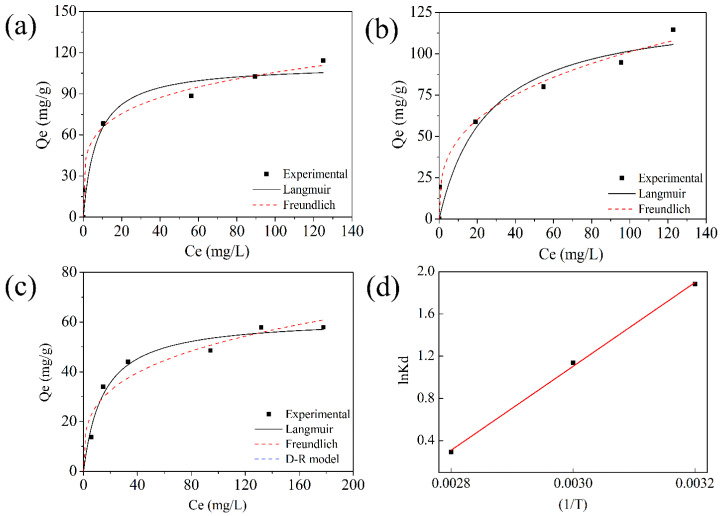
Adsorption isotherms for Sr^2+^ at 313 K (**a**), 333 K (**b**), and 353 K (**c**); (**d**) Van ’t Hoff plot of Sr^2+^ adsorption onto UiO-66-(COOH)_2_ at temperature of 313, 333, and 353 K (initial concentration 80 mg L^−1^, pH = 5.0, contact time 5 h).

The fitting sorption isotherms based on Langmuir and Freundlich models are shown in Figure 3a–c, and related parameters are presented in Table 2. A total of 1/n is the constant indicative of the intensity of the sorption process. Once the n value is greater than 1, it indicates a strong interaction between the surfaces of the adsorbent and adsorbate. Thus, it is still a chemisorption domination process under various temperature ranges. The sorption process conducted at 353 K can be better fitted with the Langmuir model. However, at lower temperatures (313 and 333 K), the Freundlich model seems to be more befitting with the experimental data. The different isotherm models under various temperatures suggest the active sites on the adsorbent surface become uneven with the decreased temperature [40]. As a result, the UiO-66-(COOH)_2_ tends to sorb Sr^2+^ at lower temperature, which is consistent with the Sr^2+^ sorption capacities of UiO-66-(COOH)_2_ at various temperatures (see Figure 3 and Table 2). It is worth noting that the maximum adsorption capacity can reach approximately 110 mg g^−1^ at 313 K, much higher than other carboxyl modified MOFs materials as compared with reported similar adsorbents listed in Table 3. This is again ascribed to this strong Sr^2+^ complexing ability of carboxylate in the framework.

To further provide a quantitative description of the adsorption behaviors, the thermodynamic parameters standard enthalpy change, entropy change, and Gibbs energy change were calculated from the temperature-dependent adsorption isotherm. The adsorption capacity decreases with the increase of temperature. The so-called Van ’t Hoff plot between lnK_d_ and 1/T, where T ranges from 313 to 353 K, is illustrated in Figure 3d, and the parameters were calculated using equations below, where K_d_ is the distribution coefficient.

(8) $$\ln K_{d}=\frac{\Delta S}{R}-\frac{\Delta H}{\mathrm{RT}}$$

(9) ΔG= ΔH−TΔS

In the case of Sr adsorption, the negative ΔH (−31510.06 J K^−1^ mol^−1^) and ΔS (−89.85 J K^−1^ mol^−1^) values, combined with negative values of ΔG (−5.49 kJ mol^−1^~−2.16 kJ mol^−1^) from 313 to 353 K suggested not only the chemisorption as confirmed above, but also a spontaneous process. Therefore, the Sr^2+^ loaded process is again proved to be the displacement of the proton, and then the strong host–guest chemical interaction with carboxylate in the framework.

This strong coordination interaction-controlled adsorption process of Sr^2+^ into UiO-66-(COOH)_2_ inspired us to explore the removal capacities of this MOF in the presence of competing ions possessing similar radius sizes. The Sr adsorption was tested in the presence of Na^+^ and K^+^ mixture with the molar ratio of 1:1:1 (Sr/Na/K). Obviously, Sr adsorption capacity exhibited a high percentage removal of 72%, easily surpassing the removal efficiency of disturbing ions (Na^+^ 3.2% and K^+^ 3.2%, respectively) (Table 2).

**Figure 4:**
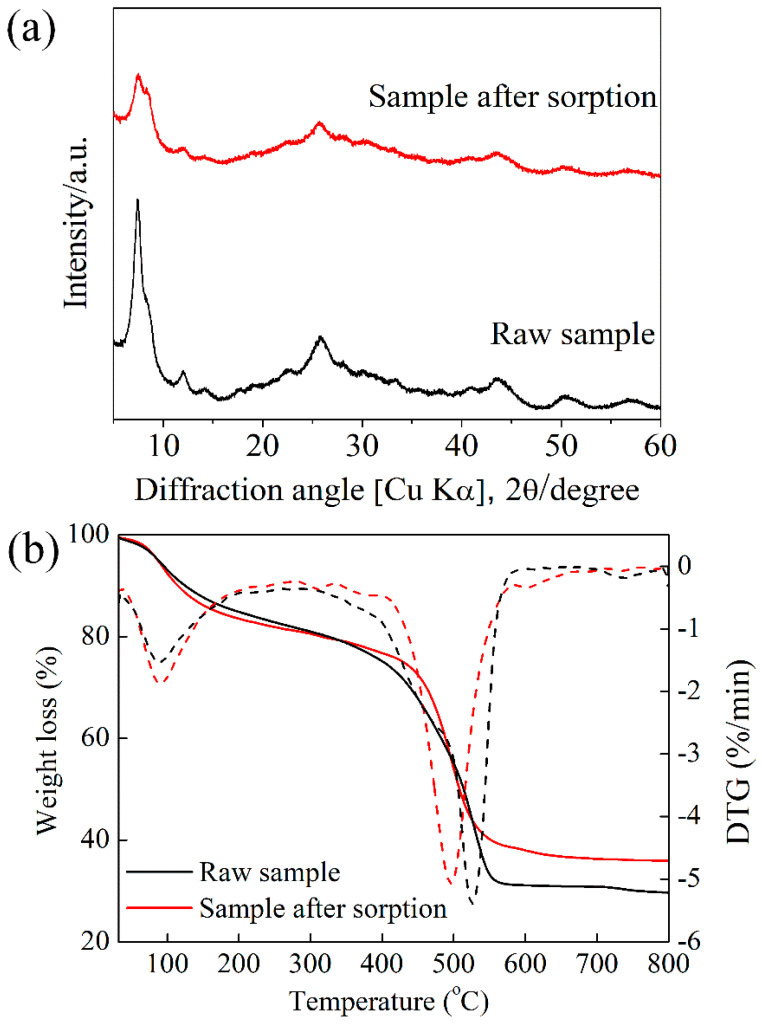
PXRD patterns (**a**) and TGA-DTG analysis (**b**) of UiO-66-(COOH)_2_.

**Figure 5:**
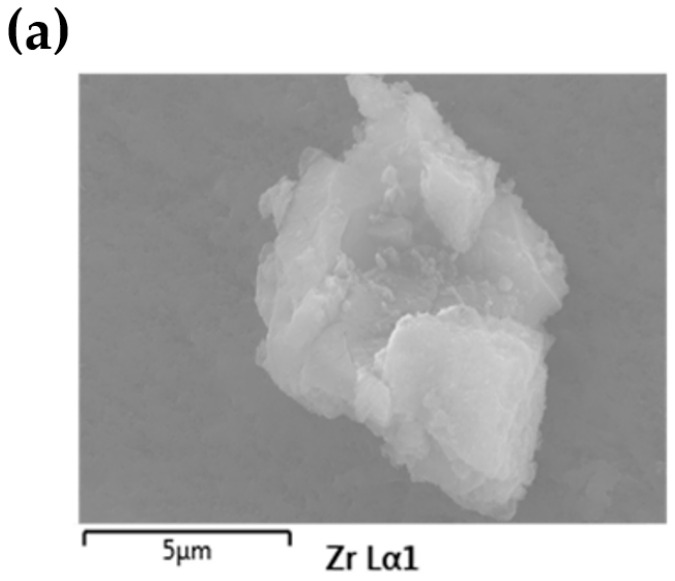

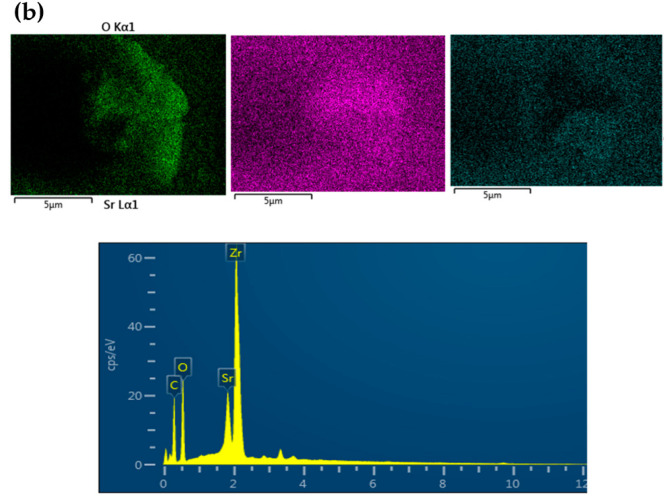
SEM images (**a**) and EDS mapping (**b**) of UiO-66-(COOH)_2_ after Sr^2+^ adsorption.

**Figure 6:**
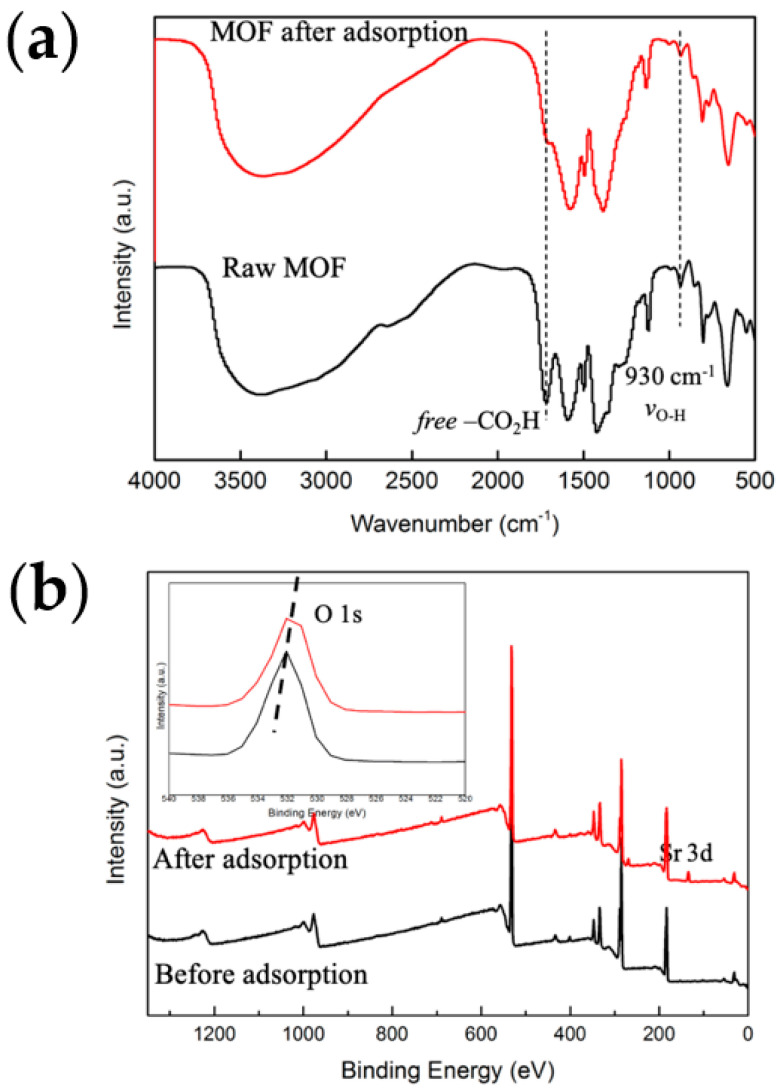
FTIR spectra (**a**) and XPS analysis (**b**) of UiO-66-(COOH)_2_ and after Sr^2+^ loading.

To understand the structural changes that occur upon the adsorption process as compared with pristine MOF, PXRD was conducted, shown in Figure 4a. It is clear from PXRD analysis that the diffraction lines of MOFs without changes in position were observed, indicating the preservation of the crystallinity. Besides, no precipitation happens to the sorption process. Additional thermal stability confirmation from TGA-DTG measurement is shown in Figure 4b. By the end of the measurement, the residue after adsorption was 6.2% higher as compared with pristine UiO-66-(COOH)_2_ corresponding to the Sr-loading. The SEM images (Figure S1) reveal that the adsorbent before and after adsorption measurement was agglomerate composed of faceted and polydistributed crystals, and can maintain either its surface or morphology [25]. EDS analysis (Figure 5) detected the four main elements C, O, Zr, and Sr, and they were well-dispersed in the particles. Especially, the content of 5.9% adsorbed Sr (Table S3) was consistent with the TGA analysis. On the other hand, we performed additional FTIR and XPS characterizations. FTIR spectrum provides evidence of deprotonation and coordination of -COOH (Figure 6a). The peak at 930 cm^−1^ is due to O-H bending vibration of the free carboxyl group. Note that this peak reduces in intensity after adsorption, and well documented that deprotonation of O-H groups. At 1710 cm^−1^, moreover, the relative intensity of the peak decreased significantly after adsorption, which is assigned to the coordination of free carboxyl groups with adsorbed ions. XPS measurement can furtherly detect the existence of Sr^2+^ after adsorption, the characteristic peak of Sr3d appeared at 134 eV. Shown in Figure 6b, the O1s peak of the carboxylic group slightly shifts from 531.98 to 531.48 eV, indicating the binding interaction between Sr and carboxyl on UiO-66-(COOH)_2_. The above characterizations are consistent with the kinetic and thermodynamic studies that the Sr^2+^ adsorbed by the stable UiO-66-(COOH)_2_ was through a coordination adsorption process with deprotonated carboxyl as the active sites.

## 4. Conclusions

The framework of UiO-66-(COOH)_2_ features non-bonded carboxylic groups, high stability, and porosity, which makes it available to adsorption of Sr ion selectively and effectively. The adsorption process involved a slow diffusion with maximum sorption attained in 5 h, at pH 5 and temperatures ranging from 298 to 353 K. The negative values of ΔG (−5.49 kJ mol^−1^~−2.16 kJ mol^−1^) indicate that the sorption Sr^2+^ on MOF was spontaneous at studied conditions. The PSO model and Langmuir model confirm the specific coordination interactions between carboxylic and Sr^2+^. Moreover, this chemical complexation in the absence of electrostatic attractions is responsible for the selectivity adsorption in the presence of disturbing ions. These strong interactions between Sr^2+^ and well-distributed -COOH on the surface also makes the adsorption capacity reach as high as 114 mg g^−1^. Our present studies provide the fact that this material shows both selectivity and high stability in the cationic mixture, and makes it a promising candidate in real-life applications.

## Figures and Tables

**Table 1:** Kinetics model parameters of Sr^2+^ adsorbed onto UiO-66-(COOH)_2_ (initial concentration 100 mg L, pH = 5.0, T = 293 K, contact time 24 h).

Kinetic Model	Parameter	Value
Pseudo-first-order	qe, exp (mg g^−1^)	42.16
	qe, cal (mg g^−1^)	38.75
	k_1_ (min^−1^)	0.264
	R^2^	0.953
Pseudo-second-order	q_e, cal_ (mg g^−1^)	40.09
	k_2_ (min^−1^)	0.753
	R^2^	0.973

**Table 2:** Calculated Langmuir and Freundlich parameters for the sorption of Sr^2+^ onto UiO-66-(COOH)_2_.

Kinetic Model	Parameter	313 K	333 K	353 K
Langmuir model	Qm (mg g^−1^)	111.361	127.475	61.747
	b (L mg^−1^)	0.140	0.039	0.068
	R^2^	0.934	0.935	0.978
Freundlich model	*n*	4.791	3.096	3.480
	K_f_ (mg g^−1^)	40.386	22.847	13.727
	R^2^	0.995	0.988	0.936

**Table 3:** Sr^2+^ adsorption data for reported carboxyl functionalized MOFs.

Carboxyl Functionalized MOFs	Adsorption Capacity (mg g^−1^)	Ref.
Zr-MOF-COOH-SO_4_	67.5	[35]
Nd-BTC-MOFs	58	[20]
1D-Ni-MOF/GO membrane	78	[41]
[Me_2_NH_2_][In(TDC)_2_]^-^1.5DMA^-^1.5H_2_O	48.83	[42]
UiO-66-(COOH)_2_	114	This work

## Data Availability

Data Availability Statements in section “MDPI Research Data Policies” at https://www.mdpi.com/ethics.

## Supplementary Materials

The following supporting information can be downloaded. Table S1: The adsorption capacity (qe) and the monitored pH values after adsorption under various pH conditions; Table S2: Zeta potential values measured of the adsorbent before and after adsorption; Table S3 The weight percentage (%) of elements detected by EDS of UiO-66-(COOH)_2_ after Sr^2+^.
