# Supplementary material for: The Carboxyl Functionalized UiO-66-(COOH)2 for Selective Adsorption of Sr2+
Source: Molecules. 2022 Feb 11;27(4):1208. doi: 10.3390/molecules27041208 (PMC8879016; doi:10.3390/molecules27041208)
Supplement: Supplementary file 1 [file molecules-27-01208-s001.zip › molecules-1577844-supplementary.pdf]

molecules

The Carboxyl Functionalized UiO-66-$(COOH)_2$ for Selective Adsorption of Sr^{2+}

Yuan Gao [1], Yinhai Pan [1,2], Zihan Zhou [1], Quanzhi Tian [2] and Rongli Jiang [1,*]

[1] School of Chemical Engineering and Technology, China University of Mining & Technology, Xuzhou 221116, China
[2] National Engineering Research Center of Coal Preparation and Purification, China University of Mining and Technology, Xuzhou, 221116, China
* Correspondence: ronglij@cumt.edu.cn

Table S1. The adsorption capacity (qe) and the monitored pH values after adsorption under various pH condition.

pH	qe (mg g^{-1})	pH after Adsorption
2	4.53	2.36
5	5.91	5.08
7	4.94	3.62

Table S2. Zeta potential values measured of the adsorbent before and after adsorption.

	Zeta Potential (mV)
UiO-66-$(COOH)_2$	-31.7
After Sr^{2+} adsorption	-33

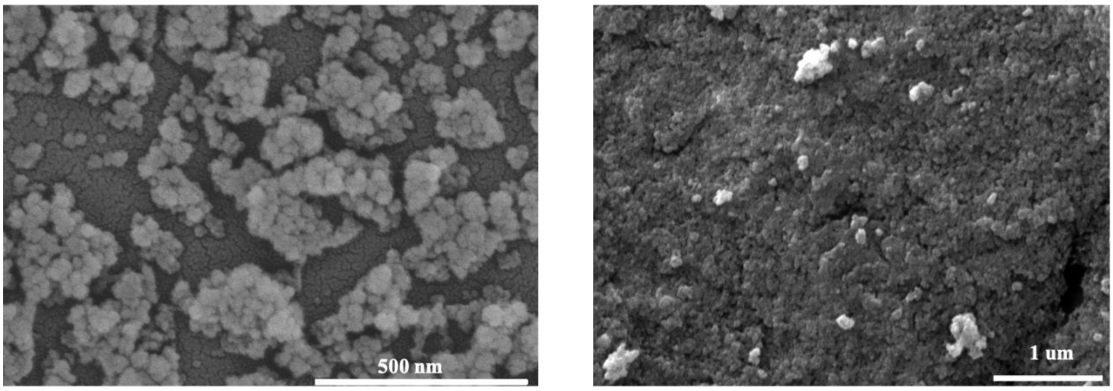

Figure S1. SEM of UiO-66-$(COOH)_2$ before (Left) and after (Right) Sr^{2+} adsorption.

Table S3. The weight percentage (%) of elements detected by EDS of UiO-66-$(COOH)_2$ after Sr^{2+} adsorption.

Element	Weight Percentage (%)
C	32.16
O	21.92
Sr	5.92
Zr	39.99
Total (%)	100
